# Comparison of medicine availability measurements at health facilities: evidence from Service Provision Assessment surveys in five sub-Saharan African countries

**DOI:** 10.1186/1472-6963-13-266

**Published:** 2013-07-09

**Authors:** Yoonjoung Choi, Paul Ametepi

**Affiliations:** 1 Office of Population and Reproductive Health, Bureau for Global Health, US Agency for International Development, 1201 Pennsylvania Avenue, NW, Suite 200, Washington, DC 20004, USA; 2 MEASURE DHS, ICF International, 11785 Beltsville Drive, Suite 300, Calverton, MD 20705, USA

**Keywords:** Medicine availability, Survey methodology, Health facility assessment, Health systems

## Abstract

**Background:**

With growing emphasis on health systems strengthening in global health, various health facility assessment methods have been used increasingly to measure medicine and commodity availability. However, few studies have systematically compared estimates of availability based on different definitions. The objective of this study was to compare estimates of medicine availability based on different definitions.

**Methods:**

A secondary data analysis was conducted using data from the Service Provision Assessment (SPA) – a nationally representative sample survey of health facilities – conducted in five countries: Kenya SPA 2010, Namibia SPA 2009, Rwanda SPA 2007, Tanzania SPA 2006, and Uganda SPA 2007. For 32 medicines, percent of facilities having the medicine were estimated using five definitions: four for current availability and one for six-month period availability. ‘Observed availability of at least one valid unit’ was used as a reference definition, and ratios between the reference and each of the other four estimates were calculated. Summary statistics of the ratios among the 32 medicines were calculated by country. The ratios were compared further between public and non-public facilities within each country.

**Results:**

Across five countries, compared to current observed availability of at least one valid unit, ‘reported availability without observation’ was on average 6% higher (ranging from 3% in Rwanda to 8% in Namibia), ‘observed availability where all units were valid’ was 11% lower (ranging from 2% in Tanzania to 19% in Uganda), and ‘six-month period availability’ was 14% lower (ranging from 5% in Namibia to 25% in Uganda).

**Conclusions:**

Medicine availability estimates vary substantially across definitions, and need to be interpreted with careful consideration of the methods used.

## Background

With growing emphasis on health systems strengthening in global health [1,2], health facility assessment methods are increasingly being used to assess readiness of facilities to deliver quality services, an aspect of complex health systems. Measuring the availability of essential medicines at facilities is one of the core components of these assessments. However, while surveys focusing on the affordability of medicines tend to follow standardized methodologies [3-5], health facility assessments have employed a wide variety of tools and approaches. Availability of commodities, including medicines, is defined differently in various facility assessment surveys. For example, whereas reported availability by respondents without verification has been used in approaches geared toward rapid assessment [6-8], in-depth facility assessment methods validate the reported response by observing the medicines, verifying the expiration dates [9,10], and collecting further data on stock-out over an extended period [10].

Few studies, however, have systematically compared estimates based on different definitions of availability. Understanding the magnitude and pattern of differences among various estimates is important for interpreting facility assessment results. The purpose of this study is to compare estimates of medicine availability based on different definitions, using data from Service Provision Assessment surveys (SPA) conducted in five sub-Saharan countries: Kenya, Namibia, Rwanda, Tanzania, and Uganda.

## Methods

### Data

SPA is a national-level survey of formal sector health facilities, providing comprehensive data on availability of services at facilities, readiness of facilities to provide essential health services, and quality of care. SPA is conducted as part of the MEASURE Demographic and Health Surveys (DHS) project, supported by the US Agency for International Development [11]. Standardized methodologies and instruments are used, providing comparable data across time and countries. As with other surveys under the project, MEASURE DHS provides technical assistance to host country implementing partners to conduct the assessment, ensuring data quality and comparability, and SPA data are freely available to the public [11].

SPA covers selected technical elements (e.g., family planning, maternal and child health, HIV/AIDS, malaria) as well as cross-element topics, such as infrastructure, infection control, and human resources at facilities. Typically, SPA is conducted as a nationally representative sample survey of facilities – including both public and non-public sector facilities and ranging from primary health care facilities to tertiary-level hospitals. The sample design allows calculation of sub-national estimates of indicators by sector, facility level, and first-level administrative unit. In a small number of countries with a limited number of facilities, a facility census approach has been used [12].

SPA utilizes four types of data collection tools: facility inventory, health worker interview, observation of consultations, and exit interviews with clients. Facility inventory tool collects facility-level information related to infrastructure, infection management procedures, laboratory diagnostic capacity, management practices, and availability of equipment, commodities and essential medicines. To assess the availability of the over 110 medicines listed in the inventory questionnaire, surveyors interview the facility staff who is in charge of medicines on the day of the assessment. For each medicine on the list, the facility staff is first asked if that medicine is stocked at the facility and available that day. For the medicines that are stocked and reported to be available, surveyors ask to see them. Formulation is specified for each medicine, but any observed strength or dosage is coded as available. In each country, brand names for the medicines are used as appropriate.

Surveyors then verify that at least one unit of each medicine in stock has a valid expiration date. Given the broad scope of SPA, it is not practical to verify the expiration date of all observed units for each of the over 110 medicines, particularly in large facilities. However, surveyors conducted a spot check by verifying expiration dates on all observed units for 32 sentinel medicines, including basic medicines to treat infectious diseases prevalent in low-resource settings (Table 1).^a^ For each medicine for which at least one valid unit is observed, surveyors ask the facility staff if the facility has experienced any stock outs of the medicine at any time during the six months preceding the survey. Reported stock-out responses are not verified against medicine registers or other records.

**Table 1:** List of the select 32 medicines for which detailed availability data were collected in SPA

**Medicine**	
1	Amoxicillin [oral]
2	Amoxicillin [injection]
3	Ampicillin [injection]
4	Ampicillin [oral]
5	Ceftriaxone [injection]
6	Chloramphenicol [oral]
7	Chloramphenicol [injection]
8	Doxycycline [oral]
9	Erythromycin [oral]
10	Kanamycin [injection]
11	Ketoconazole [oral or topical]
12	Loperamide [oral]
13	Miconazole [vaginal suppository]
14	Norfloxacin [oral]
15	Nystatin [oral]
16	Nystatin [vaginal suppository]
17	Oral rehydration salts
18	Penicillin Benzyl [injection]
19	Penicillin, procaine [injection]
20	Penicillin-V [oral]
21	Phenobarbital [oral or injection]
22	Sulfadiazine [oral]
23	Artemisinin [oral]
24	Artemether-Lumefantrin [oral]
25	Sulfadoxin + Pyrimethamine [oral]
26	Quinine [oral]
27	Quinine [injection]
28	Chloroquine [oral]
29	Chloroquine [injection]
30	Amodiaquine [oral]
31	Rringers lactate [injection]
32	Plasma expander [injection]

Data for this secondary analysis came from recent SPA conducted in five sub-Saharan African countries: Kenya SPA 2010, Namibia SPA 2009, Rwanda SPA 2007, Tanzania SPA 2006, and Uganda SPA 2007. A nationally representative sample of 695, 611, and 491 facilities were assessed in Kenya, Tanzania, and Uganda, respectively. In Namibia data were collected from all 411 formal sector facilities. In Rwanda, data were collected from all public facilities and a sample of private facilities, and a total of 538 health facilities were assessed.

### Measurement and analysis

For the 32 medicines for which expiration dates were verified on all observed units, four dichotomous variables were constructed to measure current (i.e., on the day of assessment) availability: reported availability without observation or verification of expiration dates – the most inclusive definition; observed availability of at least one unit regardless of expiration dates; observed availability of at least one valid unit; and observed availability where all units are valid. For each medicine, percent of facilities with the medicine were calculated using the four different definitions of current availability. ‘Observed availability of at least one valid unit’ was used as a reference definition of current availability for two reasons: i) it is the most restricted definition routinely collected for all medicines assessed in SPA beyond the selected 32 medicines; ii) it is also the definition currently used in SPA final reports. In order to assess relative differences between the reference estimate and each of the other three estimates, a ratio of the comparison to the reference estimate was calculated.

In addition, a binary variable was constructed to measure retrospective six-month availability for the 32 medicines: ‘Observed availability of at least one valid unit’ on the day of survey (i.e. reference definition of current availability) and no history of stock-out during the six months before the survey. The stock-out history was based on respondents’ report. For each medicine, percent of facilities with the medicine were calculated using the six-month period availability. In order to assess the relative difference compared to the reference current availability, a ratio of the six-month period to the current availability estimate was calculated.

All analyses were conducted by country. Summary statistics of the ratios were calculated among the 32 medicines. We further compared distributions of the ratios by managing authority (public vs. non-public) and facility level (primary, health center, and hospitals), using *T*-test. Non-public facilities include facilities managed by private, non-governmental organization, and faith-based organizations. Small sample sizes limited analyses by further breakdown of these facilities. Classification by level followed country-specific definitions and categories provided in each of the final SPA reports. Primary facilities are front line formal health facilities where communities seek basic ambulatory services and typically include – but not limited to – clinics, dispensaries, and health posts (see Table 2 for detailed country-specific information).

**Table 2:** Percent distribution of facilities by managing authority and level: by country

	**Kenya**	**Namibia**	**Rwanda**	**Tanzania**	**Uganda**
	**(n = 695)**	**(n = 411)**	**(n = 486)**	**(n = 602)**	**(n = 479)**
Managing authority					
mPublic	49.7	74.5	62.1	67.6	76.0
Non-public*	50.3	25.6	37.9	32.4	24.0
Total	100.0	100.0	100.0	100.0	100.0
Level					
Hospital	7.3	11.0	8.6	4.1	9.5
Health center	11.5	11.4	77.6	9.1	32.2
Primary†	81.3	77.6	13.8	86.8	58.3
Total	100.0	100.0	100.0	100.0	100.0

Estimates were adjusted for sampling weights in Kenya, Tanzania, and Uganda.

* Non-public facilities include facilities managed by private, non-governmental organization, and faith based organizations.

† Classification of facilities by level followed country-specific definitions and categories provided in each SPA. Primary care facilities included: clinics, dispensaries, maternity, and stand-alone VCT clinics in Kenya SPA 2010; clinics, free standing VCT, and sick bays in Namibia SPA 2009; dispensaries, health posts, and clinics in Rwanda SPA 2007; dispensaries and stand-alone VCT clinics in Tanzania SPA 2006, and health center level II in Uganda SPA 2007.

A small number of facilities that typically do not store medicines were excluded from all analyses (52 in Rwanda, 9 in Tanzania, and 12 in Uganda). Further, in Tanzania and Uganda, 1.7% and 5.2% of sampled facilities had multiple pharmacies or medicine storage rooms. For those, a medicine was categorized to be available if it was available in either pharmacy or medicine storage room. Finally, since SPA was a sample survey in Kenya, Tanzania, and Uganda, all estimates were adjusted for sampling weight in these countries. STATA 11.0 statistical software (Stata Corporation, College Station, TX, USA) was used for the analysis.

## Results

Table 2 presents basic characteristics of facilities included in the analysis. The majority of facilities were primary care facilities or health centers. The overall percent of facilities that were public varied from 50% in Kenya to 76% in Uganda. Across levels, public facilities were the majority in most countries (results not shown). In Rwanda, however, where only 14% of facilities were reported to be primary-level facilities, 73% of primary-level facilities were non-public facilities.

**Figure 1:**
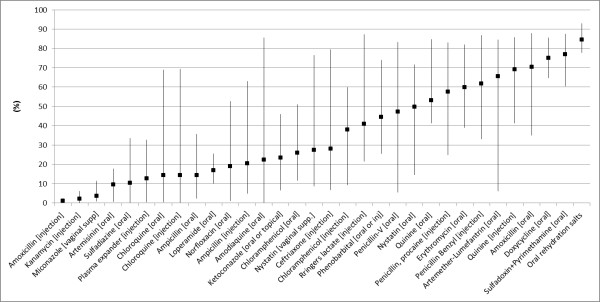
**Current availability of the select 32 medicines in the five study countries: percent of facilities with at least one valid unit observed on the day of survey.** Square is a mean estimate among the five surveys. Vertical lines represent a range for each medicine.

Current availability based on the reference definition – observed availability of at least one valid unit (hereafter referred to as reference availability), varied greatly across most of the medicines and countries (Figure 1). Nevertheless, a small number of medicines showed relatively similar levels across countries. In all five countries, the availability was lower than 20% for Amoxicillin injectible, Kanamycin, Miconazole, and Artemisinin, while it exceeded 60% in Doxycycline, Sulfadoxin/Pyrimethamine, and oral rehydration salts (Figure 1).

Table 3 presents the average of the reference availability estimates among the 32 medicines by country. Average availability across the 32 medicines ranged from 32.3% in Namibia to 44.2% in Rwanda. However, again, availability varied greatly among the 32 medicines within each country.

**Table 3:** Estimates of current availability (%) of 32 medicines based on the reference definition*: by country

	**Mean**	**Median**	**SD**	**Min**	**Max**
Kenya	35.4	31.9	26.9	0.8	83.5
Namibia	32.3	21.3	32.1	0.3	93.1
Rwanda	44.2	49.9	34.1	0.2	87.2
Tanzania	36.4	22.4	33.6	0.2	87.6
Uganda	33.1	25.6	29.5	0.0	87.3

*Reference definition: at least one valid unit observed at the facility on the day of survey.

Among the 32 medicines, compared to the reference value, reported availability without observation or verification of expiration dates was higher on average by 3% in Rwanda and by 8% in Namibia (Table 4), with an un-weighted average of 6% among the five countries. Observed availability of at least one unit regardless of expiration dates was higher than the reference values on average by 0.4% in Rwanda and by 7% in Namibia, with an un-weighted average of 4% among the countries. There was larger cross-country variation in relative differences between estimates based on the most restricted definition (i.e., all observed units are valid) and the reference values. Availability based on the restricted definition was lower than the reference value by only 2% in Tanzania but 19% in Uganda.

**Table 4:** Relative differences in estimates compared to reference estimates* among 32 medicines: by country

	**Ratio: comparison-to-reference**						
**Comparison definition**	**Country**	**n†**	**Mean**	**Median**	**SD**	**Min**	**Max**
Current availability							
Reported							
	Kenya	32	1.062	1.016	0.121	1.002	1.503
	Namibia	32	1.078	1.026	0.194	1.000	2.000
	Rwanda	32	1.032	1.009	0.065	1.000	1.273
	Tanzania	32	1.061	1.011	0.169	1.000	1.846
	Uganda	31	1.055	1.010	0.180	1.000	2.000
Observed, at least one unit regardless of validity							
	Kenya	32	1.054	1.013	0.122	1.000	1.503
	Namibia	32	1.071	1.009	0.195	1.000	2.000
	Rwanda	32	1.004	1.000	0.016	1.000	1.091
	Tanzania	32	1.052	1.002	0.171	1.000	1.846
	Uganda	31	1.009	1.000	0.036	1.000	1.196
Observed, all units valid							
	Kenya	32	0.900	0.973	0.245	0.000	1.000
	Namibia	32	0.909	0.976	0.242	0.000	1.000
	Rwanda	32	0.853	0.856	0.069	0.719	1.000
	Tanzania	32	0.981	0.985	0.019	0.924	1.000
	Uganda	31	0.815	0.831	0.095	0.498	1.000
Six-month period availability							
	Kenya	32	0.864	0.853	0.067	0.742	0.986
	Namibia	32	0.951	0.960	0.060	0.667	1.000
	Rwanda	32	0.859	0.908	0.171	0.000	1.000
	Tanzania	32	0.870	0.877	0.097	0.568	1.000
	Uganda	31	0.748	0.762	0.172	0.000	1.000

SD: standard deviation.

*Reference value: percent of facilities with at least one valid unit observed on the day of survey.

**†**In Uganda, the reference estimate was zero for one medicine and statistics are among 31 medicines.

**Figure 2:**
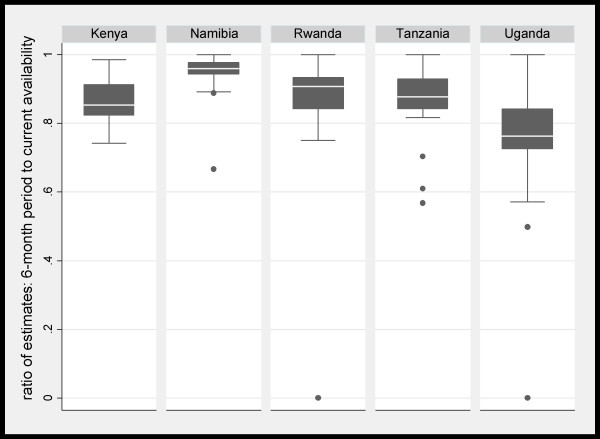
Boxplot of ratios of six-month to current* availability estimates among 32† medicines: by country. Note: Interquartile range: 0.03 in Namibia; 0.09 in Kenya, Rwanda, and Tanzania; and 0.12 in Uganda. Reference value: percent of facilities with at least one valid unit observed on the day of survey. † In Uganda, the reference estimate was zero for one medicine and statistics are among 31 medicines.

Retrospective six-month period availability was lower than the reference definition of current availability by a wide range of magnitude across countries and medicines. Among the 32 medicines, the six-month availability estimates were lower than the current availability estimates on average by 25% in Uganda but only by 5% in Namibia (Table 4). Further, within each country, there was a substantial range of relative differences between the six-month period and current estimates among the 32 medicines, except in Namibia (Figure 2).

By managing authority, in all countries except Uganda, there were no statistically significant differences in reference current availability (Table 5) or relative differences in current availability among the 32 medicines (Table 6). In Uganda, however, current availability of the 32 medicines was 28.1% in public facilities and 49.1% in non-public facilities (Table 5), and current availability based on the most restricted definition (i.e., all observed units are valid) was on average 26% lower than the reference value in public facilities – significantly larger than the difference of 13% in non-public facilities. Finally, in Kenya, Tanzania, and Uganda, public facilities had significantly larger differences than non-public facilities in relative differences between six-month availability and current availability among the 32 medicines (Table 6).

**Table 5:** Estimates of current availability of 32 medicines*: by country and managing authority (%)

**Country**	**Sector**					**Level**							
	**Public**		**Non-public**			**Primary**		**Health center**			**Hospital**		
	**Mean**	**SE**	**Mean**	**SE**	**p-value†**	**Mean**	**SE**	**Mean**	**SE**	**p-value†**	**Mean**	**SE**	**p-value†**
Kenya	33.3	5.6	37.7	4.6	0.55	33.2	4.6	40.3	5.6	0.33	50.9	5.9	0.02
Namibia	33.2	6.1	28.8	4.5	0.56	27.2	5.8	36.1	6.1	0.29	61.9	6.5	<0.001
Rwanda	46.1	6.5	41.1	5.4	0.55	22.9	3.6	46.0	6.5	<0.001	61.8	6.7	<0.001
Tanzania	33.5	6.4	42.5	5.6	0.30	35.3	6.0	39.0	5.8	0.65	55.1	6.6	0.03
Uganda	28.1	5.2	49.1	5.8	0.009	30.8	5.3	33.9	5.2	0.68	45.1	5.4	0.06

SE: standard error.

*Reference definition: at least one valid unit observed at the facility on the day of survey.

† P-value for *t*-test of distributions of estimates between public and non-public facilities. The number of observations is 32 in all subgroups.

**Table 6:** Relative differences in estimates compared to reference estimates* (ratio) among 32 medicines: by country and managing authority

**Definition**	**Country**	**Managing authority**							**Level**										
		**Public**			**Non-public**				**Primary**			**Health center**				**Hospital**			
		**n†**	**Mean**	**SE**	**n†**	**Mean**	**SE**	**p-value‡**	**n†**	**Mean**	**SE**	**n†**	**Mean**	**SE**	**p-value‡**	**n†**	**Mean**	**SE**	**p-value‡**
Current availability																			
Reported																			
	Kenya	32	1.36	0.24	32	1.02	0.01	0.17	32	1.09	0.04	31	1.02	0.01	0.06	32	1.01	0.00	0.04
	Namibia	30	1.09	0.05	32	1.03	0.01	0.20	30	1.08	0.03	26	1.02	0.01	0.12	32	1.01	0.00	0.03
	Rwanda	32	1.04	0.01	31	1.02	0.01	0.42	26	1.03	0.01	32	1.05	0.02	0.34	30	1.01	0.01	0.01
	Tanzania	31	1.08	0.06	32	1.02	0.00	0.26	31	1.07	0.04	30	1.01	0.00	0.09	31	1.03	0.01	0.33
	Uganda	30	1.04	0.02	31	1.04	0.03	0.96	29	1.02	0.01	30	1.04	0.01	0.21	31	1.05	0.03	0.39
Observed, at least one unit regardless of validity																			
	Kenya	32	1.35	0.24	32	1.02	0.00	0.17	32	1.09	0.04	31	1.00	0.00	0.04	32	1.00	0.00	0.04
	Namibia	30	1.08	0.05	32	1.02	0.01	0.19	30	1.07	0.03	26	1.02	0.01	0.25	32	1.00	0.00	0.06
	Rwanda	32	1.00	0.00	31	1.01	0.00	0.26	26	1.01	0.00	32	1.01	0.00	0.60	30	1.00	0.00	0.03
	Tanzania	31	1.07	0.06	32	1.01	0.00	0.27	31	1.06	0.04	30	1.00	0.00	0.13	31	1.01	0.01	0.22
	Uganda	30	1.02	0.01	31	1.00	0.00	0.37	29	1.01	0.01	30	1.01	0.01	0.92	31	1.01	0.01	0.84
Observed, all units valid																			
	Kenya	32	0.87	0.05	32	0.89	0.05	0.74	32	0.89	0.05	31	0.91	0.04	0.78	32	0.90	0.04	0.85
	Namibia	30	0.91	0.05	32	0.92	0.04	0.91	30	0.89	0.05	26	0.86	0.05	0.63	32	0.92	0.04	0.69
	Rwanda	32	0.87	0.01	31	0.83	0.02	0.19	26	0.86	0.03	32	0.85	0.01	0.70	30	0.88	0.01	0.36
	Tanzania	31	0.99	0.00	32	0.98	0.00	0.60	31	0.98	0.00	30	0.99	0.01	0.16	31	0.99	0.00	<0.001
	Uganda	30	0.74	0.03	31	0.87	0.02	<0.001	29	0.81	0.03	30	0.78	0.04	0.55	31	0.82	0.01	0.65
Six-month period availability																			
	Kenya	32	0.76	0.03	32	0.90	0.01	0.00	32	0.86	0.01	31	0.89	0.02	0.16	32	0.87	0.01	0.39
	Namibia	30	0.94	0.02	32	0.97	0.01	0.15	30	0.92	0.03	26	0.93	0.02	0.70	32	0.98	0.00	0.05
	Rwanda	32	0.85	0.03	31	0.89	0.02	0.29	26	0.86	0.04	32	0.87	0.03	0.88	30	0.91	0.02	0.26
	Tanzania	31	0.80	0.04	32	0.91	0.01	0.01	31	0.86	0.02	30	0.87	0.02	0.81	31	0.90	0.02	0.17
	Uganda	30	0.70	0.03	31	0.82	0.03	0.01	29	0.81	0.02	30	0.74	0.03	0.09	31	0.78	0.03	0.43

SE: standard error.

*Reference value: percent of facilities with at least one valid unit observed on the day of survey.

† n is less than 32, when reference estimates were zero for one or more medicines among the 32.

‡ P-value for *t*-test of ratio distributions compared public facilities or primary facilities.

By facility level, reference current availability was significantly higher in hospitals compared to that in primary facilities in most countries, although the difference was only marginally significant in Uganda (Table 5). In general, relative differences in current availability among the 32 medicines tended to be larger in primary facilities, compared to those in hospitals (Table 6).

## Discussion

Given the growing emphasis on monitoring and evaluation of health systems strengthening programs in developing countries, understanding how to interpret and compare findings from various health facility assessments has taken on added importance [1,13]. This study, a secondary analysis of SPA conducted in five sub-Saharan African countries, systematically compared estimates of medicine availability using five definitions of availability (four of current availability and one six-month availability). Our results show that estimates of current availability vary substantially depending on how it is defined. Compared to ‘observed availability of at least one valid unit’, the reference definition used in this study, availability of medicines based solely on reported response was on average 6% higher across the five countries, while observed availability where all units were valid was 11% lower. In addition, availability during the six-month period preceding the survey was 14% lower than the reference. The pattern and magnitude of relative differences in various current availability estimates were comparable between public and non-public facilities in most countries. However, in Kenya, Tanzania, and Uganda, relative differences between the six-month availability and reference values were significantly larger among public facilities than non-public facilities.

Definitions of current availability used in our study provide rather simple snapshots of medicine availability. The reference definition of ‘observed availability of at least one valid unit on the day of assessment’ does not reveal the presence of expired medicines in the storage area, which would indicate poor commodity supply management practices. Neither the reference nor the more strict definition of current availability (i.e., observed availability in which all units are valid) provide any information on whether facilities currently have a sufficient amount of medicines on-hand to meet client needs. Also, current availability may not be a good proxy for availability over an extended period, as our results showed a wide range of variation in six-month period to current availability ratios. Finally, affordability and rational use of medicines, as well as the presence of falsified and substandard medicines, are important aspects of pharmaceutical systems that cannot be assessed from availability alone.

Nevertheless, in a large scale health facility assessment such as SPA – with a large sample size to provide sub-national estimates of indicators across a number of services, we believe ‘observed availability of at least one valid unit’ is the most appropriate definition to measure current medicine availability. The reference definition has practical advantages compared to the other definitions assessed in this analysis. First, compared to the two more inclusive definitions of current availability (i.e., reported availability, and observed availability without verifying validity), it provides more accurate data, while requiring minimal additional costs in fieldwork. One of the most important factors determining implementation cost is the total number facilities selected and, especially in low-resource settings where transportation can be limited, the number of facilities that can be visited per day. Once the survey team is at a sampled facility, the additional time required to observe medicines and verify the expiration date of one unit per medicine is typically not long enough to affect implementation costs. And, compared to the more strict definition of current availability (verification of the expiration date of every unit), the reference definition can reduce surveyor fatigue substantially – especially in large facilities, which is critical for achieving high data quality. Finally, the six-month period availability definition has limited value, since it is based on reported responses on ever having stock-out. In order to measure period availability with minimum errors and bias, medicine registers need to be reviewed for all medicines to obtain the number of clinic days during the period and presence of medicines on each clinic day. While it is important for facilities to be able to provide such detailed information for supply chain management purposes, reviewing such data would not be feasible for a large-scale health facility assessment.

Balancing these practical advantages and limitations, the SPA questionnaires have been recently revised to include only questions that are necessary to calculate estimates based on the reference current availability defition [10]. Questions regarding verification of all units for selected medicines and reported six-month stock out were eliminated. The revised questions to assess medicine availability have been adopted in the World Health Organization’s latest health facility assessment tool, the Service Availability and Readiness Assessment (SARA) [14].

There are limitations in our analysis. First, considering potential recall errors in reporting retrospective six-month stock-out, the relative difference of 11% between the current and the six-month period availability estimates might have been underestimated. Second, relative differences across estimates in our study, the fairly small difference between the reported and the observed current availability in particular, may be limited to surveys collecting both reported and verified responses. In SPA, surveyors inform the respondents that they will validate expiration dates of available units for each medicine. Thus, respondents have little incentive to provide systematically biased responses; therefore, any difference between reported and observed availability is likely due to random reporting errors. However, in assessments that rely on reported responses only, there may be more reporting bias in addition to random errors, depending on the objective and purpose of the assessment. For example, if a health facility assessment is conducted for monitoring and evaluation of performance based financing programs, there may be increased incentives to underreport stock-outs. Finally, in comparison of results by managing authority and facility level, the small number of medicines could have contributed to lack of statistical power in spite of relatively large differences between sub-groups in some cases. Also, if all or a portion of the 32 medicines are supplied by the same distributor, availability among them might be correlated, violating an assumption of independence among observations for *T*-test.

## Conclusions

In summary, estimates of medicine availability vary substantially depending on definitions. Observed availability of at least one valid unit may be an appropriate definition to be used in a large scale health facility assessment, considering feasibility and cost of assessments and the limitations of other definitions. To interpret and compare results from various facility assessments, users need to consider the methods and definitions carefully.

## Endnotes

^a^Country-specific guidelines may vary in terms of whether these medicines are required at all health facilities. However, considering the burden of diseases in low-resource settings, most facilities are expected to have the 32 medicines.

## Abbreviations

DHS: Demographic and Health Surveys; SARA: Service Availability and Readiness Assessment; SPA: Service Provision Assessment Survey.

## Competing interests

The authors do not have a commercial or other association that might pose a conflict of interest.

## Authors’ contributions

YC contributed to study design, data analysis, interpretation of results, and manuscript writing. PA contributed to interpretation of results, and manuscript writing. Both authors read and approved the final manuscript.

## Pre-publication history

The pre-publication history for this paper can be accessed here:

http://www.biomedcentral.com/1472-6963/13/266/prepub
